# *Mycobacterium avium *complex immune reconstitution inflammatory syndrome: Long term outcomes

**DOI:** 10.1186/1479-5876-5-50

**Published:** 2007-10-15

**Authors:** James Riddell, Daniel R Kaul, Petros C Karakousis, Joel E Gallant, Jennifer Mitty, Powel H Kazanjian

**Affiliations:** 1 Department of Internal Medicine, Division of Infectious Diseases, University of Michigan Health System, Ann Arbor, Michigan, USA; 2 Department of Medicine, Division of Infectious Diseases, Johns Hopkins University School of Medicine, Baltimore, Maryland, USA; 3 Department of Internal Medicine, Division of Infectious Diseases, Brown University, Providence, Rhode Island, USA; 4 Methodist Hospital, Indianapolis, Indiana, USA

## Abstract

**Background:**

To describe long term outcomes of *Mycobacterium avium *complex (MAC) immune reconstitution inflammatory syndrome (IRIS).

**Methods:**

Cases of MAC IRIS were retrospectively identified at four HIV clinics (Michigan, Maryland, Rhode Island, and Indiana) from 1996–2004. Patients were included if they were initially diagnosed with AIDS and found to have evidence of focal MAC infection documented by tissue culture or PCR after initiating HAART, and at least 6 months of follow up.

**Results:**

Among the 20 patients included, the mean age was 40 years, mean CD4 cell count was 24/mm^3 ^at pretreatment baseline and 100/mm^3 ^at time of MAC IRIS diagnosis. Sites of disease included lymph nodes (15 patients [8 peripheral, 8 abdominal and 1 peripheral and abdominal]), gastrointestinal tract (7) and liver (3). Sixteen patients (80%) responded to treatment and were disease free after a mean of 17.4 months of therapy for MAC IRIS; IRIS therapy was withdrawn in 6 without relapse. Four patients (non-responder group) had persistent or relapsing disease despite 27 months of ongoing MAC IRIS treatment. At the time of resolution or last follow-up, the mean CD4 cell count and viral load was 143/mm^3 ^and 7,000 c/mL for responders, and 65/mm^3 ^and 17,000 c/mL for non-responders, respectively. Most patients with peripheral adenopathy were responders (7/8; 88%); many with abdominal adenopathy (4/8; 50%) were nonresponders.

**Conclusion:**

The majority of patients with MAC IRIS eventually responded to treatment. Our sample size was not adequate to perform statistical analysis, but there was a tendency towards adequate CD4 response to HAART and peripheral rather than intraabdominal adenopathy among responders.

## Background

Focal manifestations of *Mycobacterium avium *complex (MAC) may occur in AIDS patients with severe immune suppression after starting HAART [1,2]. This is thought to be the result of specific cell mediated immune response to MAC antigens associated with highly active antiretroviral therapy (HAART). The syndrome, referred to as the immune reconstitution inflammatory syndrome (IRIS), most often occurs within 3 months of initiating HAART, but may also occur years later. The development of IRIS may reflect an unmasking of subclinical disease or worsening of the intensity or alteration of the manifestations of MAC infection that had been present prior to initiation of HAART [3,4].

Studies have addressed the clinical features of IRIS at onset, as well as the course of six patients after one year [5-10] and 29 months of follow up [11]. Other reports have described patients with refractory or relapsing MAC IRIS disease [5,7,9,10]. Nonetheless, it remains unknown whether the majority of patients with this syndrome will eventually respond to specific treatment, and what features may accompany successful response to therapy. We undertook this study to better define the long-term outcome of MAC IRIS.

## Methods

Patients who received treatment at four university HIV/AIDS programs in the United States (University of Michigan, Ann Arbor, MI; Brown University, Providence, RI; Johns Hopkins University, Baltimore, MD; Methodist Hospital, Indianapolis, IN) were retrospectively identified from 1998 to December 2004. One patient (#18) has been previously reported elsewhere [10]. We used a previously described definition of IRIS for criteria for inclusion [3]; specifically, patients were included if they were initially diagnosed with AIDS, were started on therapy with HAART, and developed focal end organ (including lymph node) MAC infection documented by culture of a normally sterile site or by PCR and histopathologic changes of tissue inflammation. Time of diagnosis after starting HAART was not used as criteria for inclusion as previously published case definitions did not include this parameter. Patients were included if they had been followed for at least 6 months after initiation of treatment for MAC IRIS. CD4 cell count and quantitative HIV viral load measurement at baseline, as well after beginning HAART, were recorded. Clinical information on site of MAC IRIS involvement and treatment were abstracted from patient records. Patients were categorized as responders if they had clinical resolution and radiological improvement of IRIS. Those who did not have evidence of resolution of IRIS or had relapsing disease despite appropriate treatment were deemed non-responders. Clinical and laboratory data were analyzed using calculation of means and standard deviations. This study was approved by the Institutional Review Board of each participating center.

## Results

### Overall patient population

Table 1 outlines the clinical features of the twenty patients with MAC IRIS that were identified (11 Michigan, 2 Rhode Island, 6 Maryland, 1 Indiana). Two additional patients were excluded from analysis because they had MAC isolated from only bone marrow in the absence of other organ involvement. Seventeen patients were male; 12 were Caucasian, 7 African-American, and one Hispanic. Risk factors for the acquisition of HIV included homosexual contact (10), heterosexual contact (4), injection drug use (4), occupational injury (1), and unknown (1). The average age was 40 years (± 8.5, range 27 to 59). Average total follow-up time was 3.8 years (range 6 months to 7 years). All patients were treated with HAART regimens that contained at least 3 drugs, including either a protease inhibitor or non-nucleoside reverse transcriptase inhibitor with two nucleoside reverse transcriptase inhibitors. The mean pre-HAART CD4 cell count was 24 ± 45/mm^3 ^which increased to 100 ± 85/mm^3 ^at the time of MAC IRIS diagnosis. In 6 patients (30%), HAART was initiated after disseminated MAC was diagnosed (mean time from MAC diagnosis to HAART initiation was 36 days ± 39). Seven of the 20 total patients had been receiving MAC prophylaxis prior to the development of MAC IRIS.

**Table 1:** Summary of clinical parameters

Characteristic	Responders N = 16	Non-responders N = 4
Age	40	38
Ethnicity:		
Caucasian	8	3
African-American	7	1
Hispanic	1	0
Gender (male)	13	4
MAC prophylaxis	4	3
Prior disseminated MAC	4	4
Time to IRIS (mean)	248 days	195 days
IRIS site*:		
Lymph node(s)	11	4
GI tract	5	2
Liver	3	0
IRIS therapy†:		
2 drugs	8	2
3 drugs	6	0
4 or more drugs	2	2
Time to resolution or follow up (mean)	522 days	888 days

* Patients may have had more than one site of IRIS involvement.

†2 drugs – macrolide + ethambutol, 3 drugs – addition of rifabutin, 4 or more drugs – addition of amikacin, fluoroquinolone, or linezolid.

The median time from initiation of HAART to diagnosis of MAC IRIS was 2.6 months (range 10 days to 4.7 years). Seven (35%) patients developed IRIS less than 60 days after initiation of HAART, 10 (50%) from 60 days to one year, and 3 (15%) after more than a year. Presenting symptoms of IRIS included fever (15 patients), abdominal pain (9), symptomatic lymphadenopathy (6), night sweats (4), loose stools (4), nausea and vomiting (4), fatigue, weight loss, and rash (1 each). The most common focal site of IRIS was lymph nodes (15 patients [8 peripheral, 8 abdominal and 1 peripheral and abdominal]). Other sites included: gastrointestinal tract (7 patients) and liver (3). All patients in whom MAC was isolated from cultures of bone marrow also had focal inflammation of another site (liver or gastrointestinal tract), reflecting the case definition of IRIS. Other sites of MAC isolation included liver biopsy (3 patients), and aspirates or biopsies of other tissues. Isolator blood cultures were positive in 2 patients who also had MAC isolated from a focal site of inflammation (Table 1). One patient in whom MAC was not isolated from cultures had a diagnosis of MAC confirmed by PCR of a lymph node specimen.

All patients received a macrolide (azithromycin or clarithromycin) and ethambutol for the treatment of MAC IRIS. Ten patients received rifabutin as an additional agent. Four patients received ciprofloxacin and two received amikacin as part of their regimen of treatment. Eight patients received therapy with adjunctive agents: 8 received oral corticosteroids for an average of 17 ± 14.6 months, and 2 received granulocyte-colony stimulating factor (G-CSF). Table 2 outlines the key clinical features of each patient.

**Table 2:** Summary of immunologic and virologic parameters (expressed as means).

	**Baseline**		**IRIS Diagnosis**		IRIS F/U*	
	**CD4**	**VL**	**CD4**	**VL**	**CD4**	**VL**

Responders (n = 18)	29 ± 50	422 K ± 502 K	88 ± 65	59 K ± 186 K	143 ± 109	7 K ± 23 K
Non-Responders (n = 4)	6 ± 4	259 K ± 185 K	152 ± 143	1 K ± 1 K	65 ± 53	17 K ± 35 K

*For responders, F/U = Resolution

### Responders

Sixteen of the 20 patients (80%) experienced clinical and/or radiographic resolution of signs of active infection after 19.5 ± 21.7 months (range 22 days to 5.2 years) of MAC IRIS treatment (patients 1–16 in Table 2). The mean pre-HAART CD4 cell count in the responder group was 29/mm^3 ^and was 88/mm^3 ^at the time of IRIS diagnosis (Table 3). The mean CD4 cell count was 143/mm^3 ^at the time of clinical resolution for the responder group (IRIS follow up in Table 3). One of the patients in this group had a relapse of infection one year after resolution of initial symptoms. Peripheral lymph node was the most common site of involvement in responders (7/16; 44%); of the eight patients with peripheral lymph node involvement, seven (88%) were responders. MAC IRIS treatment has been withdrawn in 6 of the 16 responders without recurrence of symptoms. Six patients from this group required adjunctive corticosteroids for a mean of 12.8 ± 5.9 months. Two cases of patients who responded successfully to treatment of MAC IRIS are described in detail below (patients #2 and #3 from Table 2).

**Table 3:** Summary of clinical and laboratory findings for Responders (1–16) and Non-responders (17–20).

						CD4 count (cells/ml)					Treatment	
#	Age, sex	Symptoms	Organ involvement	Diagnostic procedure	Baseline viral load^a^	Baseline^b^	At time of IRIS	At time of follow up^c^	Viral load at follow up	Time to IRIS^d ^(days)	MAC therapy	Duration of corticosteroid (months)

1	37, F	Fever, N/V	GI, liver	Liver biopsy	249,880	10	54	84	<50	226	Azm, ETB	12
2	40, M	Fever, abd pain	Abd LN	LN aspirate	32,318	43	165	229	<50	66	Azm, ETB	---
3	44, M	Fever, abd pain	GI, Bone marrow	BMBx, blood Cx	215,201	69	199	326	<50	25	Clm, ETB, RIB, cipro	22
4	59, M	Fever, abd pain	GI, Bone marrow	GI biopsy, BMBx	188,000	34	98	140	<50	604	Azm, ETB, RIB, cipro	10
5	33, M	LAN	Axillary LN	LN aspirate	72,777	20	64	89	<50	1689	Clm, ETB	---
6	53, M	LAN	Axillary LN	Tissue culture	750,000	9	111	201	<50	306	Clm, ETB	---
7	53, M	Fever, rash, NS	Supraclavicular LN	LN aspirate	31,517	5	142	97	<50	192	Azm, ETB, RIB	---
8	47, M	Fever, abd pain, NS	Supraclavicular LN, Liver	Liver biopsy	500,000	2	3	13	21,439	122	Azm, ETB, RIB	Not available
9	32, M	LAN	Cervical LN	LN aspirate	117,580	204	224	403	<50	456	Clm, ETB, RIB	---
10	28, M	LAN	Axillary LN	Tissue culture	217,174	8	66	226	<50	75	Clm, ETB, RIB	---
11	38, M	Fever, Wt loss	Liver, Bone marrow	Liver Bx, BMBx	750,000	4	35	99	87	10	Clm, ETB	---
12	38, M	Fever, LAN, fatigue	Cervical LN	Tissue culture	366,000	16	46	10	97,000	18	Azm, ETB, RIB	---
13	40, M	Fever, abd pain, N/V, diarrhea	Abd LN, GI	LN aspirate	321,851	21	71	73	849	84	Azm, ETB	6
14	40, M	Fever, diarrhea, cough	ABD LN, Mediastinal LN	Blood Cx, CT	624,697	1	18	48	<50	63	Clm, ETB, RIB	14
15	30, F	Fever, NS, diarrhea	GI, Bone marrow	Tissue Bx, BMBx	205,115	9	39	141	336	16	Clm, ETB	---
16	42, F	Fever, NS	Abd LN	Blood Cx, CT	2,115,623	7	65	118	2,729	17	Clm, ETB	---
17	46, M	LAN, abd pain	Abd LN, supraclavicular LN, mediastinal LN	LN aspirate	292,571	11	72	108	<50	359	Azm, ETB, RIB, cipro, amikacin	---
18	44, M	Fever, abd pain	Abd LN	LN aspirate	205,000	5	61	40	<50	360	Clm, ETB, RIB, cipro, amikacin	48
19	27, M	Fever, abd pain, N/V	GI, abd LN	Blood Cx, CT	490,668	6	112	111	<50	47	Azm, ETB	7
20	35, M	Fever, abd pain, N/V, diarrhea	GI, abd LN	Blood Cx, stool Cx, CT	48,053	2	364	3	70,324	13	Azm, ETB	---

**Note: **N/V, nausea and vomiting; abd, abdominal; LAN, lymphadenopathy; NS, night sweats; GI, gastrointestinal tract; LN, lymph node; Cx, culture; BMBx, bone marrow biopsy; Bx, biopsy; CT, CAT scan findings; Azm, azithromycin; ETB, ethambutol; Clm, clarithromycin; RIB, rifabutin.

^a ^HIV quantitative PCR measured as copies/cc of plasma.

^b ^CD4 count prior to the initiation of HAART.

^c ^For responders, follow up = resolution of symptoms.

^d ^Duration of time from the initiation of HAART to the development of IRIS symptoms.

*Patient #2 *(Responder; resolution of intra-abdominal MAC IRIS lymphadenitis following MAC IRIS therapy and sustained immunologic recovery after the initiation of HAART): A 40-year-old man initially diagnosed with *Pneumocystis *pneumonia and HIV infection (CD4 cell count 43/mm^3^, viral load 32,318 c/mL) initially responded well to trimethoprim/sulfamethoxazole and corticosteroids. He then developed recurrent fevers up to 102°F after beginning zidovudine, lamivudine, and efavirenz. An abdominal CT scan showed multiple enlarged mesenteric, retroperitoneal and periaortic lymph nodes. A lymph node core needle biopsy of an enlarged lymph node showed necrotizing granulomas. *Mycobacterium avium *complex was isolated from culture of the biopsy specimen. He received azithromycin and ethambutol and continued HAART. Clinical improvement was documented within one month and radiologic resolution at one year. The CD4 cell count at that time was 229/mm^3^, and the viral load was undetectable. Antimycobacterial therapy was then discontinued.

*Patient #3 *(Responder; required prolonged corticosteroid administration to manage inflammatory complications of MAC IRIS involving the gastrointestinal tract): A 44-year-old man was initially diagnosed with *Pneumocystis *pneumonia and HIV infection with a CD4 count of 69/mm^3 ^and a viral load of 215,201 c/mL. HAART was initiated and antimycobacterial therapy was begun 2 months after an AFB isolator blood culture drawn at the time of his initial diagnosis because of ongoing fever yielded MAC. One month later, fevers persisted, and MAC was isolated from cultures of bone marrow. The antimycobacterial regimen was intensified with rifabutin and ciprofloxacin in addition to azithromycin and ethambutol, and his fever resolved. Two months later he developed recurrent fever and abdominal pain. A repeat blood culture was positive for MAC. An abdominal CT scan demonstrated multiple enlarged intra-abdominal lymph nodes. His CD4 count had increased to 199/mm^3 ^with a viral load of 684 c/mL. Because of significant abdominal pain associated with MAC IRIS involving the gastrointestinal tract, oral corticosteroids were initiated. Seven days later, his symptoms of abdominal pain completely resolved. Corticosteroids were unable to be withdrawn because of persistent abdominal pain and were therefore continued for 27 months. Antimycobacterial therapy was discontinued after 3 years of treatment.

### Non-responders

Four patients (numbers 17–20 in Table 2) had signs of persistent, focal, end organ inflammation due to MAC IRIS despite combination antimycobacterial treatment (27 ± 33 months, range 7 months to 6.3 years) and continued HAART. Of these four non-responders, two died 7 months (patient #20) and 19 months (patient # 17) after diagnosis. Patient # 20 developed rapid progression of Kaposi sarcoma with the development of ascites that became infected leading to overwhelming sepsis. Thus the clinical impact of MAC recurrence could not be evaluated in his case. A detailed description of patient #17 is outlined below. The mean CD4 cell count was 6/mm^3 ^in this non-responder group prior to treatment, 152/mm^3 ^at IRIS diagnosis, and 65/mm^3 ^at the time of last follow up (Table 3). Abdominal lymph node was the most common site of involvement in non-responders (4/4 [100%]); of the eight patients with abdominal lymph node involvement, four (50%) were responders. There was no major difference in viral load responses between the 2 groups at the time of IRIS diagnosis or IRIS follow up (Table 3).

**Figure 1:**
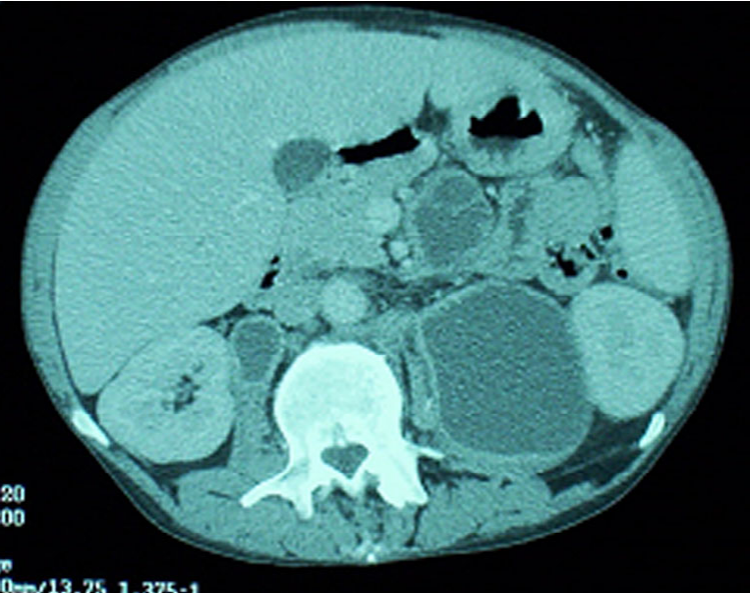
Abdominal CT demonstrating multiple abscessed intra-abdominal lymph nodes.

**Figure 2:**
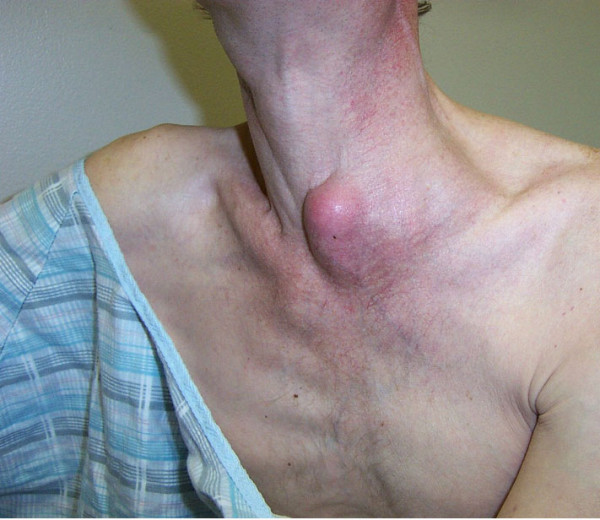
Left supraclavicular abscessed lymph node. Written informed consent was obtained for use of this image from the patient's relative.

*Patient #17 *(Non-responder; persistent MAC IRIS despite treatment with poor immunologic response to HAART): A 46-year-old man was diagnosed with HIV infection in 1989 with a CD4 cell count of 4/mm^3^. Because of alcoholism, he took antiretroviral therapy only intermittently. In 8/02 his CD4 cell count was 11/mm^3 ^and his viral load was 292,571 c/mL. A screening blood culture yielded MAC, and he was started on ethambutol in addition to azithromycin. In 9/02 he discontinued alcohol use and began HAART with a regimen of didanosine, efavirenz, and lopinavir/ritonavir. His CD4 cell count increased to 99/mm^3 ^with a reduction in viral load to 3,636 c/mL. In 9/03, with a CD4 cell count of 72/mm^3 ^and viral load of 1,991 c/mL, he was evaluated for severe right sided upper abdominal pain. An abdominal CT scan revealed multiple enlarged necrotic intra-abdominal lymph nodes (Figure 1). A percutaneous aspiration yielded 200 cc of purulent material that demonstrated many AFB organisms on smear, and culture grew MAC. The antimycobacterial regimen was intensified with the addition of rifabutin and ciprofloxacin. No information regarding macrolide susceptibility was available. Despite treatment, he developed an enlarged supraclavicular lymph node abscess on this regimen (Figure 2), which upon aspiration was found to contain MAC. Amikacin and G-CSF were then added for an additional six weeks. Because of persistent drainage from the supraclavicular lymph node, which had eroded to the surface despite percutaneous drainage, he also began a 4-week course of linezolid. Despite HAART, his CD4 count was 108/mm^3 ^with a viral load < 50 c/mL. His intra-abdominal necrotic lymphadenopathy persisted despite 15 months of treatment. He died 6 months later from complications related to squamous cell carcinoma of the rectum.

## Discussion

The majority of patients with MAC IRIS (80%) had a complete response to continuation of HAART and antimycobacterial agents over a 36 month follow up period. Of note, our study size was not large enough to permit a statistical analysis of features that may predict a favorable outcome. Furthermore, there has been a decline in incidence of MAC IRIS associated with the recent availability of potent HIV agents effective in those with late stage or highly resistant HIV infection. Because larger studies may therefore not be forthcoming, there may be a utility in making observations from our study about features that may accompany favorable responses to MAC IRIS. For example, we, as others, have noted a tendency of responders to maintain a more substantial HAART-associated CD4 cell count increase than non-responders [11]. In addition, responders tended to have involvement of peripheral rather than abdominal lymph nodes as the site of MAC IRIS. Although these observations suggest certain factors may be important in the ultimate control of MAC IRIS – CD4 responses and site of IRIS involvement, the larger controlled studies that would be necessary to confirm these observations may not be accomplished within the near future. Until then, the trends observed in our report may be useful to clinicians currently managing patients with MAC IRIS.

There has been insufficient information published in the literature regarding the characteristics of patients who do not respond to initial antimycobacterial therapy and who develop persistent MAC IRIS disease. As is the case in our study, the previous studies were unable to perform statistical analyses of prognostic factors because of small sample sizes. Of the three papers that have reported three such cases (without duplication of cases from the present study), the average CD4 cell count prior to the initiation of HAART was 96/mm^3 ^with an increase to 213/mm^3 ^at the diagnosis of IRIS [5,7,9]. Few data are available regarding the CD4 cell count at the time of relapse. One patient had a CD4 cell count of 3/mm^3^, while the other had a CD4 count of 382/mm^3^; both presented with lymphadenitis. No repeat CD4 cell count was reported for one patient with chronic MAC involving breast tissue. These patients developed MAC IRIS on average 5.6 months after starting HAART. In addition, there is insufficient data regarding whether a particular site of involvement, such as a peripheral lymph node, may have a more favorable outcome because of a lower burden of mycobacterial organisms in tissue, as well as being more amenable to surgical resection and medical management.

The lack of a standardized duration of antimycobacterial therapy required to manage IRIS reflects the variation in time in which a clinical and radiologic response may be observed. In some patients with vigorous CD4 responses to HAART, antimycobacterial agents may be withdrawn after resolution of IRIS without the development of reactivation. On the other hand, patients in our study with MAC IRIS who did not experience a significant CD4 response to HAART, who did not have improvement in symptoms, and/or who failed to eradicate MAC infection, were managed with intensification of antimycobacterial therapy and sometimes the addition of adjunctive therapy. Of note, however, the strategy of antimycobacterial therapy intensification, as well as the contribution made by adjunctive agents in the management of MAC IRIS has not been verified in controlled, prospective trials.

Our findings regarding the clinical features of MAC at the time of diagnosis are consistent with prior reports. We and others have found that IRIS is a focal inflammatory condition most often involving lymph nodes, liver or gastrointestinal tract. In addition, blood cultures rarely grow MAC at the time of IRIS; rather, a biopsy of an involved focal organ, such as lymph node, GI tract, or liver, is usually required to establish a diagnosis [3]. Furthermore, as has been shown in prior series, most (75%) but not all MAC IRIS events occurred within 3 months of initiating HAART [4,11-13]. Most patients in other series also had advanced immune suppression at the initiation of HAART (CD4 cell count 30/mm^3 ^or less) and a CD4 cell count increase in response to HAART at the time of IRIS diagnosis [4,11-13]. Finally, corticosteroids are required to manage symptoms in some, but not all patients.

In conclusion, the majority of patients with MAC IRIS experience favorable responses to long-term antimycobacterial therapy. Patients who have an adequate CD4 response to HAART may have antimycobacterial treatment safely withdrawn following resolution of focal involvement. However, neither the present study nor previous reports have had ample numbers of patients to permit statistical analyses of factors predicting a favorable outcome. Furthermore, the incidence of MAC IRIS may be declining with the development of newer agents to manage HIV. Nonetheless, the observations of the current study – that adequate immunologic responses to HAART and peripheral lymph node location of MAC IRIS may be associated with favorable outcomes, may be useful for physicians managing patients with AIDS who develop MAC IRIS.

## Competing interests

The author(s) declare that they have no competing interests.

## Authors' contributions

JR coordinated study, performed analysis of data, drafted manuscript

DRK contributed subject data

PCK contributed subject data, participated in study design

JEG contributed subject data, edited manuscript

JM contributed subject data

PHK conceived of study and participated in design, edited manuscript

All authors read and approved the final manuscript.

## References

[B1] Race EM, Adelson-Mitty J, Kriegel GR, Barlam TF, Reimann KA, Letvin NL, Japour AJ (1998). Focal mycobacterial lymphadenitis following initiation of protease-inhibitor therapy in patients with advanced HIV-1 disease. Lancet.

[B2] Phillips P, Kwiatkowski MB, Copland M, Craib K, Montaner J (1999). Mycobacterial lymphadenitis associated with the initiation of combination antiretroviral therapy. JAIDS.

[B3] Shelburne SA, Hamill RJ, Rodriquez-Barradas MC, Greenberg SB, Atmar RL, Musher DM, Gathe JC, Visnegarwala F, Trauntner BW (2002). Immune reconstitution inflammatory syndrome: Emergence of a unique syndrome during highly active antiretroviral therapy. Medicine.

[B4] Lawn SD, Bekker LG, Miller RF (2005). Immune reconstitution disease associated with mycobacterial infections in HIV-infected individuals receiving antiretrovirals. Lancet Infect Dis.

[B5] Desimone JA, Babinchak TJ, Kaulback KR, Pomerantz RJ (2003). Treatment of mycobacterium avium complex immune reconstitution disease in HIV-1 infected individuals. AIDS Patient Care STDS.

[B6] Price L, O'Mahony C (2000). Focal adenitis developing after immune reconstitution with HAART. Int J STD AIDS.

[B7] Cunningham CO, Selwyn PA (2003). Mastitis due to mycobacterium avium complex in an HIV-infected woman taking highly active antiretroviral therapy. AIDS Patient Care STDS.

[B8] DiPerri G, Bonora S, Vento S, Allegranzi B, Concia E (1998). Highly active antiretroviral therapy. Lancet.

[B9] Jenny-Avital ER (2003). A patient with refractory disseminated Mycobacterium avium after immune-reconstitution localized MAC. AIDS Clin Care.

[B10] Cinti SK, Kaul DR, Sax PE, Crane LR, Kazanjian PH (2000). Recurrence of mycobacterium avium infection in patients receiving highly active antiretroviral therapy and antimycobacterial agents. Clin Infect Dis.

[B11] Phillips P, Bonner S, Gataric N, Bai T, Wilcox P, Hogg R, O'Shaughnessy M, Montaner J (2005). Nontuberculous mycobacterial immune reconstitution syndrome in HIV-infected patients: Spectrum of disease and long-term follow-up. Clin Infect Dis.

[B12] Shelburne SA, Visnegarwala F, Darcourt J, Graviss EA, Giordano TP, White AC, Hamill RJ (2005). Incidence and risk factors for immune reconstitution inflammatory syndrome during highly active antiretroviral therapy. AIDS.

[B13] Robertson J, Meier M, Wall J, Ying J, Fichtenbaum CJ (2006). Immune reconstitution syndrome in HIV: Validating a case definition and identifying clinical predictors in persons initiating antiretroviral therapy. Clin Infect Dis.

